# Characterization of EpCAM-Positive and EpCAM-Negative Tumor Cells in Early-Stage Breast Cancer

**DOI:** 10.3390/ijms252011109

**Published:** 2024-10-16

**Authors:** Vladimir M. Perelmuter, Evgeniya S. Grigoryeva, Vladimir V. Alifanov, Anna Yu. Kalinchuk, Elena S. Andryuhova, Olga E. Savelieva, Ivan A. Patskan, Olga D. Bragina, Evgeniy Yu. Garbukov, Mariya A. Vostrikova, Marina V. Zavyalova, Evgeny V. Denisov, Nadezhda V. Cherdyntseva, Liubov A. Tashireva

**Affiliations:** 1 The Department of General and Molecular Pathology, Cancer Research Institute, Tomsk National Research Medical Center, Russian Academy of Sciences, Tomsk 634009, Russia; 2 The Laboratory of Molecular Therapy of Cancer, Cancer Research Institute, Tomsk National Research Medical Center, Russian Academy of Sciences, Tomsk 634009, Russia; 3 The Laboratory of Molecular Oncology and Immunology, Cancer Research Institute, Tomsk National Research Medical Center, Russian Academy of Sciences, Tomsk 634009, Russia; 4 The Department of Nuclear Therapy and Diagnostics, Cancer Research Institute, Tomsk National Research Medical Center, Russian Academy of Sciences, Tomsk 634009, Russia; 5 The Department of General Oncology, Cancer Research Institute, Tomsk National Research Medical Center, Russian Academy of Sciences, Tomsk 634009, Russia; 6 The Laboratory of Cancer Progression Biology, Cancer Research Institute, Tomsk National Research Medical Center, Russian Academy of Sciences, Tomsk 634009, Russia

**Keywords:** breast cancer, metastasis, circulating tumor cells

## Abstract

Most studies on CTCs have focused on isolating cells that express EpCAM. In this study, we emphasize the presence of EpCAM-negative and EpCAM^low^ CTCs, in addition to EpCAM^high^ CTCs, in early BC. We evaluated stem cell markers (CD44/CD24 and CD133) and EMT markers (N-cadherin) in each subpopulation. Our findings indicate that all stemness variants were present in both EpCAM^high^ and EpCAM-negative CTCs, whereas only one variant of stemness (nonCD44+CD24−/CD133+) was observed among EpCAM^low^ CTCs. Nearly all EpCAM^high^ CTCs were represented by CD133+ stem cells. Notably, the hybrid EMT phenotype was more prevalent among EpCAM-negative CTCs. scRNA-seq of isolated CTCs and primary tumor partially confirmed this pattern. Therefore, further investigation is imperative to elucidate the prognostic significance of EpCAM-negative and EpCAM^low^ CTCs.

## 1. Introduction

Most investigations on circulating tumor cells (CTCs) have utilized methodologies designed to isolate cells expressing EpCAM. This preference is largely attributed to CELLSEARCH being the first FDA-approved technique for detecting CD45-negative, EpCAM-positive, and cytokeratin 8-, 18-, and/or 19-positive CTCs in peripheral blood [1]. The clinical relevance of EpCAM-positive CTC detection has been substantiated in multiple studies. It was shown that an increased count of CTCs expressing EpCAM was linked to reduced overall survival across diverse cancer types [2,3]. In breast cancer, CTCs are detected in about 60% of advanced and around 20–30% of early patients [4]. The prognostic significance of EpCAM-positive CTCs in BC is associated with diminished progression-free survival and overall survival, observed in both metastatic and non-metastatic BC, as well as other metastatic cancers [5,6]. The IMENEO international meta-analysis, which analyzed over 2000 nonmetastatic BC patients from 16 centers undergoing neoadjuvant chemotherapy, revealed that the statistical significance of CTC count was rising with CTC number, from none for one cell to HR of 6.25 (95% confidence interval (CI) = 4.34 to 9.09) for five or more cells [7]. The randomized trial SUCCESS-A on more than 2000 patients revealed that CTC positivity before as well as after adjuvant chemotherapy was an independent prognostic factor, with poor disease-free survival (DFS) (hazard ratio (HR) = 2.28, 95% CI = 1.48 to 3.50) and OS (HR = 3.95, 95% CI = 2.13 to 7.32). Patients with at least 5 CTC per 30 mL showed the worst prognosis [8]. At the same time, SUCCESS-A and the ECOG-ACRIN study E5103 showed an increased risk of recurrence for patients with persisting CTCs two years and even five years after neoadjuvant chemotherapy [9,10].

The prognostic implications of EpCAM-positive expression in CTCs lack clarity across other cancer types. For example, patients with pancreatic cancer, colorectal cancer, and non-small-cell lung cancer often exhibit low numbers of these cells [11].

Elevated quantities of EpCAM-positive CTCs correlate with reduced survival in individuals with gastric cancer, as well as in the basal-like and luminal molecular subtypes of BC. Conversely, EpCAM-positive CTCs demonstrate a positive prognostic value in patients diagnosed with head and neck squamous cell carcinoma and HER2+ BC [12,13,14].

Furthermore, the expression of EpCAM in tumor cells is recognized to be linked with the presence of stemness [15]. Hiraga et al. (2016) demonstrated that BC cells expressing EpCAM exhibit a proclivity for self-renewal and differentiation, displaying heightened in vivo aggressiveness compared to their EpCAM-negative counterparts [16]. Indeed, the unfavorable prognosis associated with elevated EpCAM expression in numerous cancer types is linked to EpCAM’s capacity to modulate fundamental biological processes, including cell differentiation, proliferation, migration, and invasion [15,17]. The role of EpCAM in conferring migratory and invasive capabilities to tumor cells is, in part, attributed to its involvement in epithelial–mesenchymal transition (EMT) development. Numerous studies have documented the downregulation of EpCAM expression during the process of EMT [18,19]. Evidence suggests that during EMT, the membrane expression of EpCAM is diminished, and the protein relocates to the nucleus [20].

Nevertheless, there is a perspective suggesting that the complete suppression of EpCAM expression in CTCs is infrequent during EMT. CTCs undergoing EMT acquire an intermediate phenotype and often continue to express EpCAM [21]. Our studies confirm that a significant proportion of CTCs have features of different EMT phenotypes [22]. In such instances, some cells either entirely lose membrane expression of EpCAM or the proportion thereof is markedly diminished. This phenomenon likely elucidates the observation identified in Königsberg et al.’s (2011) study, where only 60% of metastatic cancer patients exhibited detectable CTCs [23]. The authors ascribed this outcome to the existence of EpCAM-negative CTCs [23,24]. Moreover, no data on the incidence of metastases in CTC-negative patients were found in the literature, but mortality rates in the groups of CTC-positive and CTC-negative patients (identified by CELLSEARCH) were not different according to Kirwan CC et al., 2012 [25]. Notably, in metastatic BC, the *EPCAM* gene expression was observed in 55.6% of EpCAM-negative CTCs and in 36.4% of CTCs with low EpCAM expression [26]. Several commercial platforms exist that enable the detection of EpCAM-negative CTCs [27]. These approaches included immunomagnetic CTC enrichment by antibodies against other markers, i.e., cytokeratins or methods based on physical features, i.e., size-based and quadrupole magnetic separation methods. Indeed, some research indicates that in certain cases, such as metastatic lung cancer, EpCAM-negative CTCs outnumber their EpCAM-positive counterparts [28]. Meanwhile, the relevance of such studies is beyond doubt since such cells may have the potency to form metastases, and their detection may contribute to the clarification of the prognosis and development of an optimal therapeutic strategy [24,29]. This is notably supported by data demonstrating that EpCAM^low^ and EpCAM^high^ CTCs exhibit comparable chromosomal aberrations and mutations, signifying a close evolutionary relationship. Furthermore, there is evidence that a reduction in EpCAM expression leads to the activation of EMT genes, indicating the metastatic potential of EpCAM-negative CTCs. According to Wen K.C. et al. (2018), low EpCAM expression enhanced the EMT of cancer cells and was correlated with advanced tumor stage and lymph node metastasis in endometrial carcinoma [30]. At the same time, no data have been found concerning the manifestation of stemness in CTCs with reduced EpCAM expression. Franken et al. (2023) strongly state a lack of knowledge about the prognostic significance of EpCAM^low^ CTCs and the association of their presence with EpCAM-positive CTCs [26]. Therefore, the isolation of CTCs with stem and EMT features through a method independent of membrane EpCAM expression will enable the detection of additional CTC populations with prognostic value [25].

The objective of this study was to evaluate the prevalence of EpCAM^high^, EpCAM^low^, and EpCAM-negative CTCs, along with examining the association of CTC subsets with features of stemness and epithelial–mesenchymal transition (EMT).

## 2. Results

### 2.1. Detection of EpCAM^high^, EpCAM^low^, and EpCAM-Negative CTCs in BC Patients

**Figure 1:**
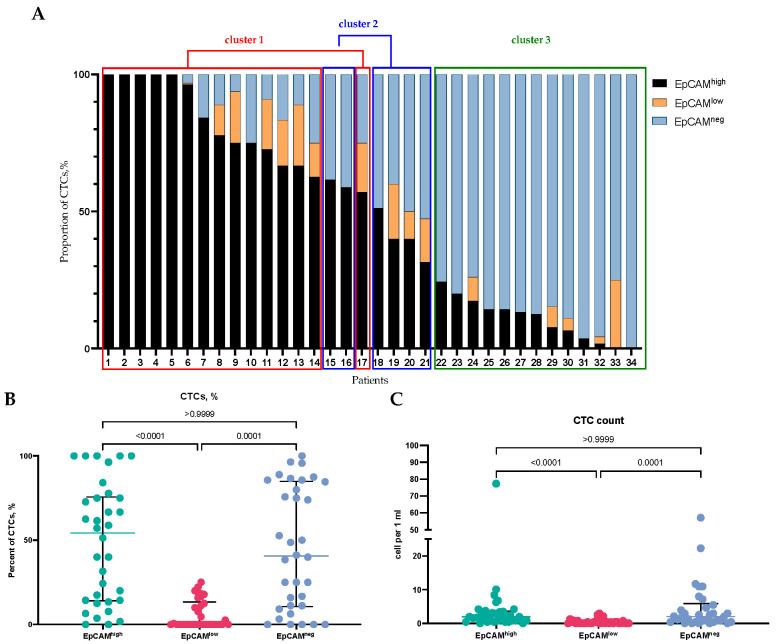
The proportion (**A**,**B**) and count (**C**) of EpCAM^high^, EpCAM^low^, and EpCAM-negative CTCs in BC patients. When determining the proportion of each CTC type, the total sum of EpCAM^high^, EpCAM^low^, and EpCAM-negative CTCs was taken as 100%.

Our study included 34 BC patients; the full clinicopathological parameters of the patients are presented in Table 1. According to the fluorescence intensity detected by flow cytometry, we categorized all CTCs based on EpCAM membrane expression into EpCAM^high^ and EpCAM^low^ subsets in each sample (Figure 1). Furthermore, we assessed the expression of cytokeratins (CKs) commonly found in breast tumor cells (cytokeratin 1, 2, 3, 4, 5, 6, 7, 8, 10, 14, 15, 16, and 19). Consequently, we were able to identify a subset of EpCAM-negative CTCs exhibiting CK expression. EpCAM^low^ cells were considered CTCs only if they showed positive expression of CKs.

It turned out that EpCAM^high^, EpCAM^low^, and EpCAM-negative CTCs were identified in almost half of patients, albeit in varying proportions. In some cases, all circulating tumor cells (CTCs) were represented by only one subpopulation, either EpCAM^high^ or EpCAM-negative cells. In other cases, a combination of three CTC phenotypes was observed (Figure 1A). The lowest proportion and count in BC patients were represented by EpCAM^low^ CTCs (*p* < 0.0001). The median count of EpCAM^high^, EpCAM^low^, and EpCAM-negative CTCs was 1.95 (0.84–3.58) cells/mL, 0.00 (0.00–0.51) cells/mL, and 2.03 (0.42–5.88) cells/mL, relatively (Figure 1B). The proportion and count of EpCAM^high^ and EpCAM-positive CTCs did not differ significantly (Figure 1B,C).

Despite the pronounced interpersonal heterogeneity, principal component analysis (PCA) allowed us to identify three clusters of patients: cluster 1—patients #1–14, 17; cluster 2—patients #15, 16, 18–21; cluster 3—patients #22–34 (Figure 1A). However, no significant differences in clinicopathological characteristics between the clusters were found (*p* > 0.05) (Table 1).

### 2.2. Detection of EpCAM^high^, EpCAM^low^, and EpCAM-Negative CTCs with Stem-like Features in BC Patients

**Figure 2:**
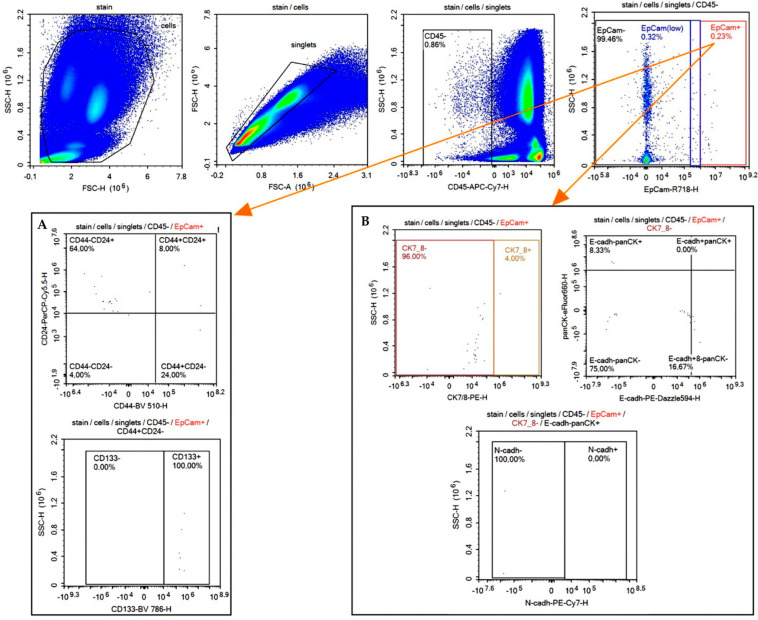
Flow cytometry analysis of EpCAM^high^ CTCs. (**A**) Evaluation of CD44/CD24 and CD133 stem markers expression. (**B**) Evaluation of epithelial cell markers cytokeratin 7/8 (CK7/8), panCK (panCK), E-cadherin, and mesenchymal cell marker N-cadherin expression in EpCAM^high^ CTCs.

We evaluated the stem features according to CD44/CD24 and CD133 expression in each of the three CTC subsets (Figure 2A).

The co-expression of studied stemness markers was found to vary among CTCs with different levels of EpCAM expression (Table 2). The frequency of occurrence of CD44+CD24−/CD133+ co-expression did not differ between EpCAM^high^ and EpCAM-negative CTCs. At the same time, CD44+CD24−/CD133− expression and the absence of all stem markers expression were less frequent among EpCAM^high^ CTCs compared to EpCAM-negative cells. Additionally, the proportion of non-stem cells was also lower in the EpCAM^high^ CTCs subset.

EpCAM^low^ CTCs with CD44+CD24−/CD133+ and CD44+CD24−/CD133− phenotypes did not occur in patients, and the frequency of EpCAM^low^ CTCs with the other two phenotypes was significantly lower than the frequency of EpCAM-negative CTCs with these two phenotypes (Table 3). When comparing the frequency of EpCAM^high^ and EpCAM^low^ CTCs, EpCAM^low^ CTCs positive for CD133, independent of CD44/CD24, were less frequent. Notably, in almost all cases, EpCAM^high^ CTCs were represented by CD133+ cells (91.2%). Moreover, not only was the frequency of detection higher, but the proportion of such cells was significantly higher among EpCAM^high^ CTCs compared to EpCAM-negative and EpCAM^low^ CTCs (Table 3).

The expression of the studied epithelial markers was minimal among the EpCAM^high^ CTCs, whereas the EpCAM-negative CTCs were characterized by a greater diversity of cells expressing CK7/8, panCK, and E-cadherin (Table 4). Only three cell subsets with N-cadherin expression were found among the studied CTC subpopulations. Within the population of EpCAM-negative CTCs, N-cadherin expression was observed only in cells without CK7/8 but with panCK expression, whereas E-cadherin expression was not significant. In the EpCAM^high^ CTCs, N-cadherin expression was observed only when no other studied epithelial markers were detected. The proportion of cells expressing N-cadherin did not differ among the EpCAM-negative, EpCAM^low^, and EpCAM^high^ CTCs.

It should be noted that more than half of the EpCAM-negative CTCs were represented by N-cadherin- cells with panCK monoexpression. The proportion of this population among EpCAM-negative CTCs was significantly higher compared to EpCAM^low^ and showed a trend-level increase compared to EpCAM^high^ CTCs (*p* = 0.0159 and *p* = 0.0637, relatively).

### 2.3. Detection of EpCAM^high^, EpCAM^low^, and EpCAM-Negative CTCs with EMT Markers in BC Patients

Next, we assessed the expression of cytokeratin 7/8 (CK7/8), E-cadherin, panCK, and N-cadherin, which are commonly altered during the EMT in EpCAM^high^, EpCAM^low^, and EpCAM-negative CTCs (Figure 2B). In our study, using the listed stemness and EMT markers, we identified 16 different phenotypic variants of cells among the EpCAM^high^, EpCAM^low^, and EpCAM-negative CTCs (Table 5). Cells with CK7/8 and panCK co-expression were absent, regardless of EpCAM expression level. EpCAM^low^ and EpCAM-negative cells lacking CK7/8 and panCK expression were not categorized as CTCs according to our criteria. However, for EpCAM^high^ cells, the lack of these epithelial markers did not preclude their classification as CTCs, provided there was pronounced EpCAM expression (4 phenotypes in EpCAM^high^ CTCs). The detection frequency of the remaining eight phenotypes in CTCs expressing EpCAM^high^ and EpCAM^low^ did not differ. The differences detected concerned the following five phenotypes: CK7/8+E-cadh−PanCK−N-cadherin−; CK7/8−E-cadh−PanCK+N-cadherin−; CK7/8−E-cadh−PanCK+N-cadherin+; CK7/8−E-cadh+ PanCK+N-cadherin−; CK7/8−E-cadh+ PanCK+N-cadherin+. The frequency of detection of the indicated CTC phenotypes was higher in EpCAM-negative cells compared to both EpCAM^high^ and EpCAM^low^ CTCs. This is evidence that CK7/8- and/or panCK expression was observed less frequently in CTCs expressing EpCAM, whereas cytokeratins were expressed more frequently in the complete absence of EpCAM membrane expression. The proportion of the investigated phenotypes did not differ significantly between the compared groups. The frequency of E-cadherin and N-cadherin expression in CTCs regardless of CK7/8 and panCK co-expression variant was higher in EpCAM-negative CTCs compared to EpCAM^high^ and EpCAM^low^ cells.

There were no differences in the frequency of occurrence of E-cadherin and N-cadherin expression between EpCAM^high^ and EpCAM^low^ CTCs, regardless of the variant of CK7/8 and panCK co-expression (Table 4). The exception was EpCAM^high^ CTCs without CK7/8 and panCK expression, where E-cadherin and N-cadherin expression were frequent.

EpCAM-negative cells with positive expression of E-cadherin and N-cadherin, as well as co-expression of these markers, were more common in patients than EpCAM^low^ cells and EpCAM^high^ cells with the same markers (Table 4).

**Figure 3:**
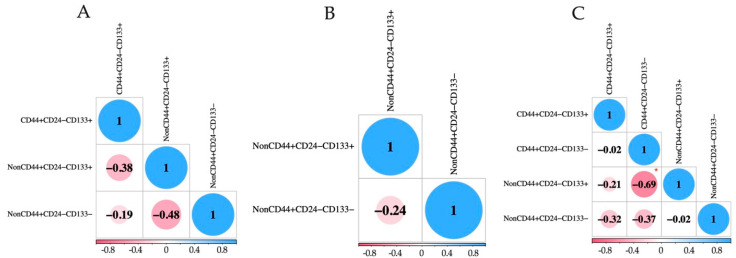
Correlation analysis between the subsets of CTCs taking into account stem features among EpCAM^high^ (**A**), EpCAM^low^ (**B**), and EpCAM-negative (**C**) tumor cells. Red asterisk indicates a significant difference (*p* < 0.05).

**Figure 4:**
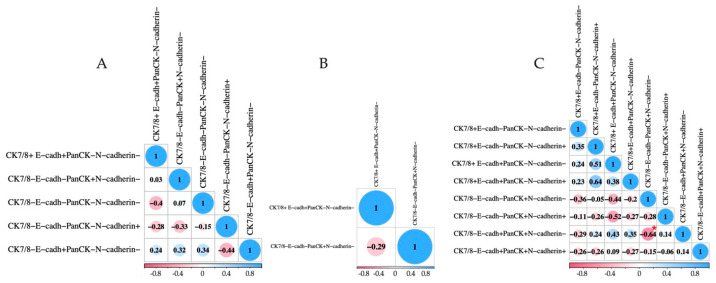
Correlation analysis between the subsets of CTCs taking into account EMT features among EpCAM^high^ (**A**), EpCAM^low^ (**B**), and EpCAM-negative (**C**) tumor cells. Red asterisk indicates a significant difference (*p* < 0.05).

Given that stem characteristics and EMT features can be reflected through various markers, the correlation of their presence on CTCs with different EpCAM expressions was assessed. We evaluated the dependence between CTC counts, taking into account stem (CD44/CD24 and CD133) and EMT features (CK7/8, panCK, E-cadherin, and N-cadherin) among each CTC subset (Figure 3 and Figure 4). We identified a negative correlation between tumor cells that were non-stem by CD44/CD24 but stem by CD133 and those that were CD44+CD24−CD133− in EpCAM-negative CTCs (r = −0.69, *p* = 0.024) (Figure 3C).

We also found a negative correlation among EpCAM-negative CTCs when considering the EMT features of tumor cells between CK7/8−E-cadherin−panCK+N-cadherin− and CK7/8−E-cadherin+panCK+N-cadherin− (r = −0.64, *p* = 0.036) (Figure 4C).

### 2.4. EpCAM^high^, EpCAM^low^, and EpCAM-Negative Tumor Cells in Primary Tumor of BC Patients

**Figure 5:**
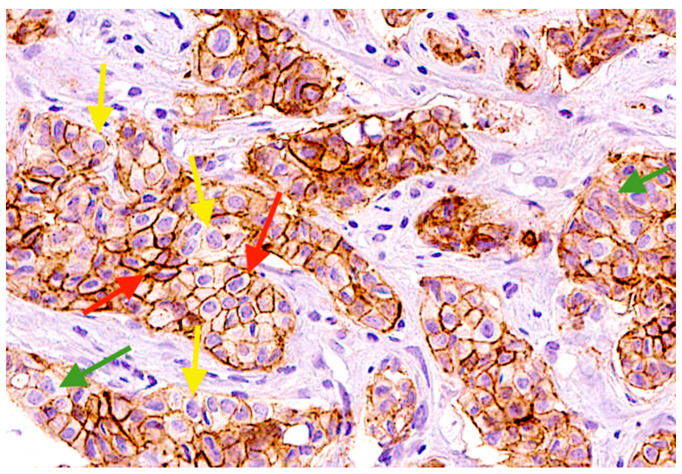
EpCAM staining of FFPE tissue slides of breast cancer patients by IHC analysis. The red arrow indicates EpCAM^high^ expression in tumor cells, the yellow arrow points to EpCAM^low^ expression, and the green arrow corresponds to the absence of EpCAM expression in tumor cells.

We conducted an immunohistochemistry analysis to evaluate the proportion of tumor cells expressing EpCAM on tissue slides obtained from 11 BC patients (Figure 5).

**Figure 6:**
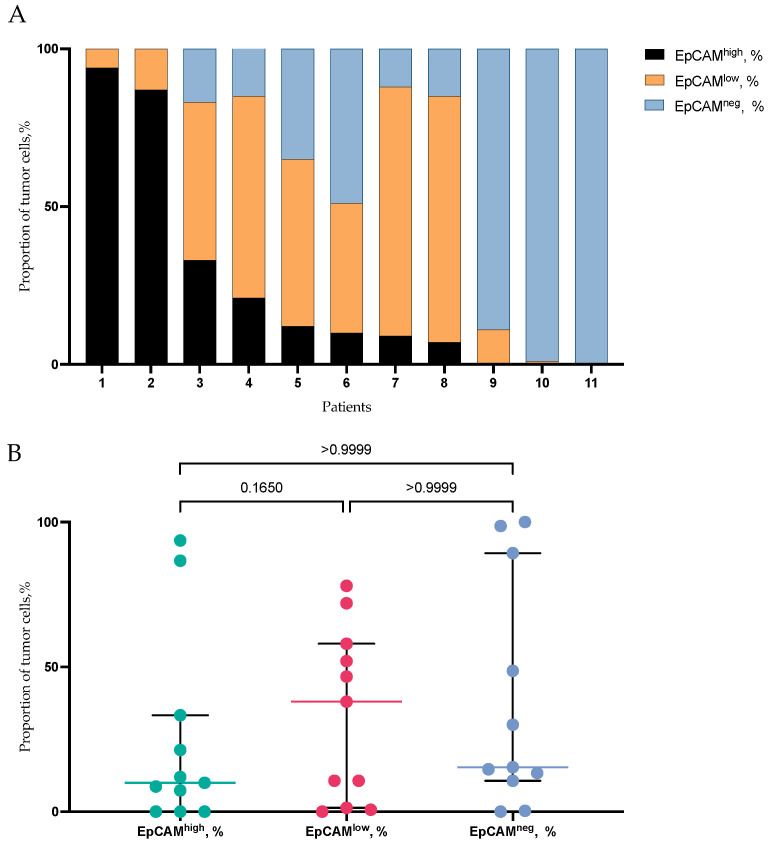
The proportion of EpCAM^high^, EpCAM^low^, and EpCAM-negative tumor cells in primary tumor of BC patients. (**A**) Interpersonal heterogeneity of EpCAM^high^, EpCAM^low^, and EpCAM-negative tumor cells in each patient. (**B**) The comparison of the proportion of EpCAM^high^, EpCAM^low^, and EpCAM-negative tumor cells in primary tumor.

Most patients displayed a combination of EpCAM^high^, EpCAM^low^, and EpCAM-negative tumor cells, while some individuals exhibited a complete absence of membrane EpCAM expression in all tumor cells, and others showed nearly total EpCAM^high^ expression (Figure 6A). In contrast to CTCs in the primary tumor, no differences in the proportion of EpCAM^high^, EpCAM^low^, and EpCAM-negative tumor cells were observed among BC patients (Figure 6B).

Comparison of the proportion and frequencies of tumor cells with EpCAM^high^, EpCAM^low^, or EpCAM-negative tumor cells among CTCs and among primary tumors showed that there was no difference between EpCAM-negative and EpCAM^high^ cells. The frequency of detection of EpCAM^low^ tumor cells and their proportion was lower among CTCs compared to primary tumors (Table 5).

We also performed a correlation analysis between the proportion of EpCAM^high^, EpCAM^low^, and EpCAM-negative cells in the primary tumor and CTCs. However, no correlation was observed between the tumor cells in any of the three subsets.

### 2.5. Transcriptomic Analysis of EPCAM-Positive and EPCAM-Negative CTCs

The total count of CTCs was determined to be 239 cells across 20 samples. *EPCAM*-negative CTCs were identified as cells with no expression of the *PTPRC* (CD45) and *EPCAM* genes, along with an expression level of epithelial genes (*KRT7*, *KRT8*, *KRT18*) exceeding 0. Conversely, *EPCAM*-positive CTCs were identified as cells lacking expression of the *PTPRC* (CD45) gene and exhibiting an expression level of the *EPCAM* gene exceeding 0. As a result, groups of *EPCAM*-negative and *EPCAM*-positive CTCs were quantified as 228 and 11 cells, respectively. We evaluated the top 100 differential gene expression in *EPCAM*-negative and *EPCAM*-positive CTCs. A full list of genes is represented in Supplementary Table S1. To identify the molecular processes activated in two groups of cells, we used the Molecular Signatures Database (MSigDB) v7.0. In *EPCAM*-negative CTCs, 52 genes were upregulated. It turned out that the highest number of overexpressed genes (5/52) was associated with allograft rejection (*p* = 3.82 × 10^−6^), while the highest number of overexpressed genes (16/97) in *EPCAM*-positive CTCS was involved in the MYC targets (V2) score (*p* = 1.64 × 10^−20^).

We utilized the approach described in our previous study [31] to investigate the stemness and EMT properties in *EPCAM*-positive and *EPCAM*-negative CTCs in each group with positive expression of stemness- and EMT-related genes (Table 6). The expression of cytokeratins was more frequently observed among *EPCAM*-negative CTCs.

Thus, *EPCAM*-positive CTCs more frequently exhibited positive expression of stemness-related genes such as ALDH1A1, CD133/PROM1, OCT4/POU5F1, and KLF4. The same pattern was revealed concerning the EMT-related genes. Specifically, among *EPCAM*-positive CTCs, cells with positive gene expression associated with various EMT variants, as indicated by *CDH1*, *VIM*, *ZEB1*, and *ZEB2* expression, were more frequently detected.

### 2.6. Spatial Transcriptomic Analysis of EPCAM-Positive and EPCAM-Negative Tumor Cells

**Figure 7:**
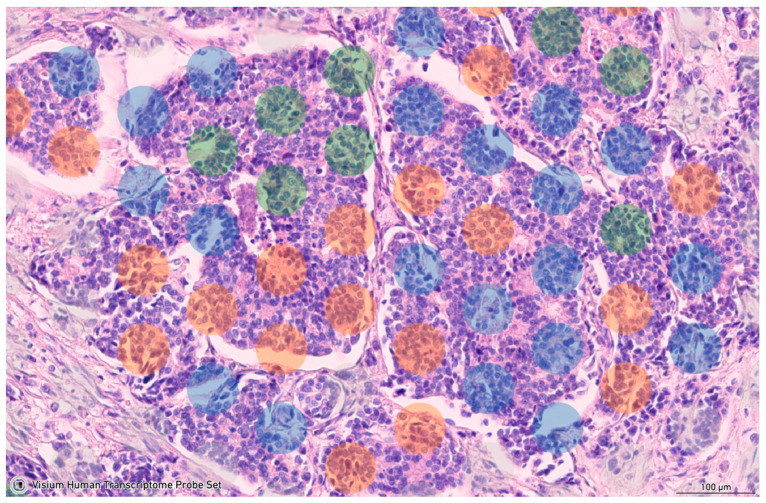
Manual annotation of spots by *EPCAM* level in BC tissue. Blue color indicates *EPCAM*-negative spots, green color—*EPCAM*^low^, and orange color—*EPCAM*^high^ spots.

Manual annotation of spots in five BC samples was conducted to identify those containing tumor cells. Spots exclusively featuring stromal cells or spots where the number of stromal cells surpassed that of tumor cells were excluded from the analysis. Subsequently, employing the Gene Filter tool, all spots were categorized into three groups based on *EPCAM* gene expression levels. Spots with *EPCAM* expression ≤ 2 units were designated as *EPCAM*-negative, spots with *EPCAM* expression ranging from 3 to 5 units were classified as *EPCAM*^low^, and spots with *EPCAM* expression ≥ 6 units were labeled as *EPCAM*^high^ (Figure 7).

We conducted pairwise differential expression analysis within the three examined BC tissue clusters. Cluster of *EPCAM*^high^ compared to *EPCAM*-negative tumor spots was characterized by upregulation of Hallmark_myc_targets_v1, GOMF_cell_adhesion_molecule_binding and GOMF_cadherin_binding pathways (3.06 × 10^−22^, 3.06 × 10^−22^, and 4.67 × 10^−16^, relatively). In the reverse scenario, in *EPCAM*-negative compared to *EPCAM*^high^ tumor spots following pathways were upregulated: Hallmark_epithelial_mesenchymal_transition, GOMF_signaling_receptor_binding, and GOBP_cell_motility (5.19 × 10^−33^, 3.66 × 10^−12^ and 3.16 × 10^−12^, relatively). Remarkably, in *EPCAM*^low^ compared to *EPCAM*-negative tumor spots, Hallmark_epithelial_mesenchymal_ transition (2.13 × 10^−50^) was also upregulated, followed by GOMF_extracellular_matrix_structural_cl_constituent and Hallmark_estrogen_response_early pathways (9.32 × 10^−31^ and 3.13 × 10^−30^, respectively).

When annotating sets of differentially expressed genes, we found that genes involved in EMT, which is required for wound healing, fibrosis, and metastasis, showed the most significant differential expression (Supplementary Table S2). Namely, these genes were found to be overexpressed in the spots with *EPCAM*-negative expression in comparison to spots with *EPCAM*^low^ and *EPCAM*^high^ expression.

Next, we manually compared the proportion of spots expressing selected gene sets among the three tissue clusters (Table 7). Despite the closeness of the numerical values of the parameters discussed, there were significant differences.

Thus, *EPCAM* ^high^ tumor spots more often demonstrated the expression of such epithelial markers as *KRT7*, *KRT8*, and *KRT18* compared to *EPCAM*^low^ and *EPCAM*-negative clusters. It is noteworthy that the *EPCAM*^high^ spots were characterized by less frequent expression of stemness-related genes such as *CD44/CD24*, *ALDH1A1*, and *SOX2*. However, the expression of *CD133/PROM1*, *OCT4/POU5F1*, *KLF4*, and *MYC* occurred more frequently compared to *EPCAM*-negative tumor spots. Additionally, the expression of *CDH1*, *SNAI1*, *SNAI2*, and *ZEB2* genes was also detected more frequently in the *EPCAM*^high^ cluster, while gene expression of *ZEB1* and *TWIST* occurred less frequently.

## 3. Discussion

A study of early-stage BC reveals that CTCs are detected in only 30% of patients [32,33]. In all the cases we studied, EpCAM-negative and EpCAM^low^ CTCs were present along with EpCAM^high^ cells. The proportion of different types of CTCs varied in each case, resulting in interpersonal heterogeneity. In the third cluster, EpCAM-negative CTCs predominated in 13 patients and were overwhelmingly EpCAM-negative. Using common methods for investigating CTCs, such as CELLSEARCH, which counts only cells with membrane EpCAM expression, these cases would have been categorized as CTC-negative. It should be emphasized that in the present study, CTCs were detected in all cases. Comparison of the three groups of patients—divided by the proportion of EpCAM^high^, EpCAM^low^, and EpCAM-negative CTCs—by molecular subtype, tumor size, grade, and lymphogenous metastasis did not reveal any differences. This indicates that, at least for the listed manifestations of tumor disease, EpCAM-negative CTCs are not less pronounced than cases with a predominance of EpCAM^high^ CTCs.

In immunohistochemical examination of primary tumors, as among CTCs, the tumor cell population was heterogeneous in terms of EpCAM expression.

However, in contrast to CTCs, no differences were found between the proportions of EpCAM^high^, EpCAM^low^, and EpCAM-negative tumor cells. However, as with CTCs, tumor cells were characterized by different combinations of the three CTC subsets, reflecting a marked interindividual heterogeneity of EpCAM expression on the membrane. This heterogeneity was manifested by EpCAM^high^ predominance in some cases and EpCAM-negative in others.

It is possible that the marked interindividual heterogeneity, manifested by different combinations of EpCAM^high^, EpCAM^low^, and EpCAM-negative tumor cells (in both primary tumors and CTCs), may be an independent criterion associated with metastasis. Notably, EpCAM^low^ and EpCAM^high^ CTCs have been described in various studies, whereas EpCAM-negative is underreported [26].

The utilization of scRNA-seq allowed us to characterize two subpopulations of CTCs: *EPCAM*-negative and *EPCAM*-positive. The evaluation of the top 100 differentially expressed genes in *EPCAM*-negative and *EPCAM*-positive CTCs clearly demonstrates the presence of completely different functional clusters. Among the 15 most expressed genes in *EPCAM*-negative CTCs, 9 genes were relevant to immune-inflammatory responses. These genes include beta 2-microglobulin, a component of the light chain of major histocompatibility complex class I (MHC I); C-C motif chemokine ligand 5 (*CCL5*); platelet factor 4 (PF4) encoding a chemokine (*CXCL4*); pro-platelet basic protein Chemokine (C-X-C motif) ligand; TSC22 domain family member 1 (*TSC22D1*)—a factor regulating TGF-beta signaling; Myosin light chain 12A, which plays a crucial role in the regulation of effector cell recruitment in foci of inflammation; and monocyte-to-macrophage differentiation-associated (*MMD*) gene. The picture is complemented by the information that five overexpressed genes are involved in the Hallmark_allograft_rejection gene set (MSigDB).

In contrast to *EPCAM*-negative, in *EPCAM*-positive CTCs, 23 overexpressed genes were associated with protein synthesis: *NPM1* (nucleophosmin 1—involved in ribosome biogenesis); *GNB2L1* receptor for activated C kinase 1 (RACK1); *PPIA* (peptidylprolyl isomerase A, CypA, which involved in protein folding and heat shock protein 90 alpha family class B member 1 regulated the tertiary structure of proteins); *EEF1B2* (eukaryotic translation elongation factor 1 beta 2—involved in the transfer of aminoacylated tRNAs to the ribosome); *HNRNPA1* (heterogenous nuclear ribonucleoprotein A1—involved in pre-mRNA packaging and 17 ribosomal protein genes, which involved in the translation process). In summary, 16 overexpressed genes were characterized by inclusion in the Hallmark_MYC_targets_v1 set, which is associated with cell proliferation, immortalization, dedifferentiation, and transformation.

Given the differentially overexpressed genes among *EPCAM*-negative CTCs, it is more likely that there are cells capable of participating in the formation of pre-metastatic niches due to their involvement in immune-inflammatory processes [34]. Among *EPCAM*-positive CTCs, there were cells with a pronounced potential for protein synthesis, which may indicate high functional activity of cells, which may include seeds that provide metastatic potential. The results of the CTCs study demonstrate a significant association between different EpCAM expression variants and stemness traits. This association is also confirmed by the work of other authors [16,35].

EpCAM overexpression was shown to promote the expression of stem cell markers (NANOG, SOX2, and OCT4) in breast cancer cell lines [36]. This occurs through translocation to the nucleus of the intracellular domain of EpICD, which is involved in modulating stemness genes, thereby maintaining cell survival [37]. However, the results suggest that the absence of membrane expression (EpCAM-negative) does not prevent the expression of stemness markers. All stemness variants (CD44+CD24−/CD133+, CD44+CD24−/CD133−, and NonCD44+CD24−/CD133+) and cells without stemness features (NonCD44+CD24−/CD133−) were found among both EpCAM^high^ and EpCAM-negative CTCs. Moreover, the frequency of CD44+CD24−/CD133− stem cells and cells without stemness features was higher among EpCAM-negative. In contrast to the stemness marker CD44+CD24−, CD133 expression was observed in CTCs regardless of the EpCAM expression variant.

The most striking difference in EpCAM^high^ CTCs is the expression of CD133 in the majority of cells (91.2%). In EpCAM^low^ CTCs, compared to EpCAM^high^ CTCs, a lower frequency of stemness variants was observed according to CD44+CD24− expression, regardless of CD133 expression. Expression of different stem markers (CD44+CD24− and CD133+), as well as their co-expression were detected more frequently in EpCAM-negative CTCs compared to EpCAM^low^ cells. In fact, only one stemness variant was observed among EpCAM^low^ CTCs, namely CD133+ but nonstem by CD44+CD24− cells.

Sequencing of single CTCs showed not only an overexpression frequency of the stemness transcription factor genes ALDH1A1, OCT4/POU5F1, and KLF4 but also, remarkably, the CD133/PROM1 gene at the protein level. In situ sequencing also confirmed a consistent pattern: in EpCAM^high^ tumor cell-dominated spots, compared to EpCAM-negative cell-dominated spots, there was a more frequent expression of the CD133/PROM1 gene along with the transcription factor genes OCT4/POU5F1, KLF4, and MYC.

Breast cancer has been shown to belong to a group of carcinomas in which EpCAM overexpression is associated with an unfavorable disease course and outcome [14]. It is likely that this is due to the more pronounced stemness features of EpCAM^high^ CTCs. The stemness of tumor cells is known to play an essential role in the progression of carcinomas, including their metastasis. What accounts for the dominance of high expression frequency of both the CD133/PROM1 gene and the CD133 protein (non-CD44+CD24−/CD133+) in the EpCAM^high^ CTC population compared to the other two stem cell subpopulations: CD44+CD24−/CD133+ (26.5%, *p* < 0.001) and CD44+CD24−/CD133− (5.9%, *p* < 0.001)? We speculate that the high percentage of stem CD133+ EpCAM^high^ CTCs may be due to the high resistance of such CTCs to cell death in the bloodstream. Previously, we found that in breast cancer, CTCs with phenotypes including co-expression of the epithelial marker CK7/8 and the stemness marker CD133, but not CD44+/CD24−, were more characterized by a lack of evidence of apoptosis. In contrast to CTCs without CD133 expression, cells expressing this stemness marker persisted in the blood after neoadjuvant chemotherapy [38]. Perhaps the association of EpCAM overexpression with poor prognosis in breast cancer is largely due to CD133 expression. It is well known that CD133 expression is associated with an increased ability to initiate tumor progression, metastasis, and recurrence in many types of cancer, including breast cancer [39,40,41].

When assessing the manifestations of EMT depending on the variant of EpCAM expression, it should first be noted that despite the controversial concept of EMT as a multilevel continuum of changes, which makes it difficult to clearly distinguish the stages [42], the consideration of protein markers allows for a cautious distinction between epithelial, hybrid, and mesenchymal phenotypes of EMT [43]. In our study, we selected the epithelial markers EpCAM, CK7/8, panCK, E-cadherin, and the mesenchymal marker N-cadherin as key markers to identify variant EMT phenotype. The earliest event of EMT is the appearance of SNAIL and ZEB1 expression; later, a switch from E-cadherin to N-cadherin expression is detected [44]. Early events in EMT also include loss in membrane expression of EpCAM and translocation of the protein to the nucleus. Many studies have reported inhibition of EpCAM expression [18]. However, studies demonstrating loss in membrane expression of EpCAM usually do not specify whether this is due to intracellular translocation or whether the cell loses EpCAM completely, as observed in our study. Loss in cytokeratin expression, along with loss in E-cadherin expression and membrane expression of EpCAM, is also associated with EMT. Huang R. et al. (2013) proposed four subgroups of EMT manifestation and believe that SNAIL is expressed in EMT at the first time point and N-cadherin, in the absence of E-cadherin, is considered as a mesenchymal sign [45].

However, it should be noted that the interpretation of the sequence of epithelial feature loss is complex and controversial. Shi R. et al. demonstrated that suppression of cytokeratin 18 (CK18) expression in breast cancer cells induces EMT and stemness through increased EpCAM expression [46]. There are attempts to categorize the different variants of EMT phenotypes into epithelial, hybrid, and mesenchymal [45,47,48]. However, the separation criteria are not sufficiently clear due to the complexity of the processes occurring during EMT-MET [36]. Loss in any of the epithelial markers in the absence of N-cadherin expression can be considered a variant of the epithelial phenotype of EMT. Any combination of the presence of epithelial features (EpCAM, E-cadherin, CK7/8, AE1/AE3) with the appearance of the mesenchymal marker N-cadherin can be considered a sign of hybrid EMT phenotypes. Evaluation of protein markers of EMT in CTCs showed that regardless of the degree of EpCAM expression (EpCAM^high^ or EpCAM^low^), expression of cytokeratins (CK7/8, AE1/AE3) was less frequent than in EpCAM-negative CTCs.

scRNA-seq of CTCs partially confirmed this pattern: expression of CK/8 and CK18 genes was less frequently observed in *EPCAM*-positive CTCs than in *EPCAM*-negative cells. In contrast to cytokeratins, the frequency of E-cadherin and N-cadherin expression in CTCs was higher in EpCAM-negative cells than in EpCAM^high^ and EpCAM^low^ cells, regardless of CK7/8 and panCK co-expression variants. Regarding the expression of transcription factor genes, the expression was higher in *EPCAM*-positive CTCs (CDH1, ZEB1, ZEB2) and in *EPCAM*-positive primary tumor cells (CDH1, SNAIL1, SNAIL2, ZEB2) compared to *EPCAM*-negative CTCs and tumor cells. SNAI1, CDH1, and FN1 gene expression was detected more frequently in *EPCAM*^low^ than in *EPCAM*-negative primary tumor spots.

Based on the above-mentioned findings, EpCAM^high^ CTCs with the loss of any cytokeratins and the absence of N-cadherin expression, independent of E-cadherin expression, can be considered variants of epithelial phenotypes of EMT. It remains unclear how these variants of epithelial phenotypes differ functionally. Since there were minimal or no EpCAM^high^ CTCs expressing N-cadherin or co-expressing N-cadherin and E-cadherin, it can be assumed that EpCAM overexpression is least associated with hybrid EMT phenotypes. Meanwhile, it is believed that hybrid EMT phenotypes have the greatest ability to initiate the development of metastases [48,49]. The opposite conclusion can be drawn regarding EpCAM-negative CTCs. EpCAM^low^ CTCs are close to EPCAM^high^ CTCs in terms of the discussed EMT markers. There is some contradiction between the expectation of aggressive EpCAM^high^ CTC potencies and the rarity of hybrid EMT phenotypes in this subpopulation.

The study has several limitations:

1. The evaluation of EpCAM expression in tumor cells, including CTCs and primary tumor cells, was conducted using different methodologies. CTCs were analyzed via flow cytometry, whereas primary tumor cells were examined through immunohistochemistry. Employing a unified approach, specifically flow cytometry, might enable a more precise comparison of EpCAM expression levels between CTCs and primary tumor cells.
2. The spectrum of epithelial markers for EpCAM-negative cells differed in immunological and genetic methods. Thus, when assessing protein expression to identify EpCAM-negative cells, cytokeratin 7/8 and pan-cytokeratin, which includes a wide range of cytokeratins (1–8, 10, 14–16, and 19 but not CK17 or CK18), were used. At the same time, in the transcriptomic analysis, only three types of cytokeratins were used: KRT7, KRT8, KRT18.
3. The evaluation of EpCAM^high^, EpCAM^low^, and EpCAM-negative CTCs presence in relation to such important clinicopathological parameters as molecular subtype, tumor size (T), and lymph node metastasis (N), which was not the aim of this study, could provide new data about the association of these cells with a worse prognosis.
4. It is known that EpCAM is associated with the proliferation potential of epithelial cells. However, our study does not cover this area of research. In this context, it would be pertinent to assess the proliferation potential of CTCs with varying levels of EpCAM expression.

## 4. Materials and Methods

### 4.1. Patients

The study involved 34 female individuals with non-metastatic invasive breast carcinoma of no special type (T1-4N0-3M0, all molecular subtypes) who received treatment at the Cancer Research Institute, Tomsk National Research Medical Center (Table 8). The study received approval from the local ethics committee of the Tomsk NRMC Institutional Review Board on 17 June 2016, with approval No. 8, and all patients granted their informed consent before participating in the analysis. The patients received treatment in accordance with the NCCN Clinical Practice Guidelines [50].

### 4.2. Flow Cytometry

Venous blood samples were collected either prior to neoadjuvant chemotherapy administration or before surgical intervention (for patients not undergoing neoadjuvant chemotherapy) using EDTA tubes. Aliquots for flow cytometry analysis (3 mL) were incubated at 37 °C for 1 h. Following incubation, plasma and white blood cells were carefully aspirated from red blood cells after sedimentation. The obtained cell concentrate was then washed with 2 mL of Cell Wash buffer (BD Biosciences, San Jose, CA, USA) through centrifugation at 800× *g* for 15 min.

CTC immunophenotyping was performed as described in our previous study [51]. Surface markers (CD45, EpCAM, N-cadherin, E-cadherin, CD44, CD24, CD133) were stained in the initial step, followed by intracellular staining during the subsequent step. The procedure began with a 10 min incubation with 5 μL of Fc Receptor-Blocking Solution (Human TruStain FcX, Sony Biotechnology, San Jose, CA, USA) at room temperature. Following this, monoclonal antibodies were added and incubated at room temperature for 20 min: BV570-anti-CD45 (clone HI30, Sony Biotechnology, USA), BV 605-anti-EpCAM (clone 9C4, Sony Biotechnology, USA), R718-anti-EpCAM (clone EBA-1, BD Biosciences, San Jose, CA, USA), PE-Dazzle594-anti-E-cadherin (clone 67A4, Sony Biotechnology, USA) PE-CF594-anti-Cytokeratin (clone CAM5.2, BD Biosciences, San Jose, CA, USA), eFluor 660-anti-pan Cytokeratin Monoclonal Antibody (panCK) (clone AE1/AE3, eBioscience, Frankfurt, Germany), PE-Cy7-anti-N-cadherin (clone 8C11, Sony Biotechnology, San Jose, CA, USA), BV 510-anti-CD44 (clone G44-26, IgG2b, BD Biosciences, San Jose, CA, USA), PerCP-Cy5.5-anti-CD24 (clone ML5, Sony Biotechnology, San Jose, CA, USA), BV 786-anti-CD133 (clone 293C3, BD Biosciences, San Jose, CA, USA). After incubation, red blood cells were lysed using 250 μL of OptiLyse C buffer (Beckman Coulter, Villepinte, France) at room temperature for 10 min in the dark, and the obtained cell suspension was washed with 1 mL of Cell Wash buffer (BD Biosciences, USA) through centrifugation at 800× *g* for 6 min.

For intracellular staining (EpCAM, CK, panCK), the cells underwent permeabilization using 250 μL of BD Cytofix/Cytoperm (BD Biosciences, USA) at 4 °C for 30 min in the dark and were subsequently washed twice in 1 mL of BD Perm/Wash buffer (BD Biosciences, USA) at 800× *g* for 6 min. The samples were then diluted in 50 μL of BD Perm/Wash buffer (BD Biosciences, USA) and incubated at 4 °C for 10 min in the dark, with the addition of 5 μL of Fc Receptor-Blocking Solution (Human TruStain FcX, Sony Biotechnology, USA). Subsequently, monoclonal antibodies were added and incubated at 4 °C for 20 min. Following incubation, the samples were washed in 1 mL of Cell Wash buffer (BD Biosciences, USA) at 800× *g* for 6 min and then diluted in 100 μL of Stain buffer (Sony Biotechnology, USA). Both unstained control and antibody quality control assessments were performed. The appropriate isotype antibodies were added to the isotype control sample at equivalent concentrations. Compensation beads (VersaComp Antibody Capture Bead kit, Beckman Coulter, Indianapolis, IL, USA) were utilized for compensation control. MCF-7 cells were used as positive control, and U937 cells were used as negative control. Flow cytometry analysis was conducted using the Novocyte 3000 (ACEA Biosciences, San Diego, CA, USA).

The gating strategy was as follows: debris and doublets were initially discriminated using forward (FSC) and side scatter (SSC) parameters, and subsequent analysis was limited by CD45-negative cells.

### 4.3. Immunohistochemical Analysis

FFPE tumor specimens were used for molecular subtype definition (*n* = 34) and evaluation of EpCAM expression by immunohistochemistry (*n* = 11). Evaluation of ERα, PR, HER2, Ki-67, and EpCAM expression was performed using Leica Bond-Max automatic immunostainer (Leica, Bannockburn, IL, USA) with Leica Refine detection kit according to standard automated protocols. Paraffin blocks were cut in a rotary microtome (Leica, Nußloch, Germany) to obtain 4 μm thick slices, which were mounted on poly-L-lysine microscope slides (Thermo Fisher Scientific, Waltham, MA, USA) and kept at 50 °C for 20 min. Next, tissue sections were deparaffinized and rehydrated according to the Leica Bond protocol. Antigen retrieval for PR antibody was performed with Bond Solution #1 (Leica Biosystems, equivalent to citrate buffer, pH 6.0), and for ERα, HER2, Ki-67, and EpCAM was performed with Bond Solution #2 (Leica Biosystems, equivalent to EDTA buffer, pH 9.0) at 100 °C for 20 min. Primary antibodies ERα(RTU, clone 6F11, mouse, Leica Biosystems, Germany), PR (RTU, clone 16, mouse, Leica Biosystems, Germany), HER2 (1:1000, polyclonal, rabbit, Dako, Carpinteria, CA, USA), Ki-67 (1:250, clone SP6, rabbit, Sigma-Aldrich, Burlington, MA, USA), and EpCAM (1:800, polyclonal, rabbit, Thermo Fisher Scientific, USA) was incubated for 25 min at RT. The primary antibody was detected with use of the Bond Polymer Refine Detection kit (Leica Biosystems, Germany) and diaminobenzidine (DAB) chromogen. Counterstaining was performed with hematoxylin. Samples were considered positive for PR and ER if at least 1% of nuclei expressed these receptors, as recommended by the American Joint Committee on Cancer. Samples were Ki-67 positive if 20% of cells expressed Ki-67. HER2 positivity was defined as at least 10% of tumor cells with a strong membrane staining score or a weak/moderate staining score accompanied by a positive fluorescent in situ hybridization result. BCs were divided into four subtypes according to ER/PR, HER2, and Ki-67 expression: luminal A (ER/PR-positive, HER2-negative), luminal B (HER2−) (ER/PR-positive, HER2-negative, Ki-67-positive), luminal B (HER2+) (ER− or PR-positive, HER2-positive), HER2+ (ER/PR-negative, HER2-positive), and triple-negative (ER/PR-negative, HER2-negative) subtypes. Epcam expression scored as negative (no staining is observed), low (strong incomplete membrane staining or weak complete membrane staining), and high (strong complete membrane staining). Based on the expression of EpCAM, the percentage of the three cell populations in the tumor sample was calculated.

### 4.4. scRNA-Seq Data Analysis of Breast CTCs

Public scRNA-seq data set from 20 BC patients (T_1-_4N_0-3_M_0_, all molecular subtypes) generated in our previous study [52] and available via BioProject under the accession number PRJNA776403 was used for investigation of transcriptional profile of *EPCAM*-negative and *EPCAM*-positive CTCs.

The Seurat software package, version 4.0.4 [53], was employed for quality control and analysis of single-cell RNA sequencing data. Cell doublets were identified using DoubletFinder [54] and subsequently removed from each dataset. Integration of the 20 datasets with default parameters was performed. The aggregated data underwent preprocessing, involving the exclusion of cells with unique feature counts less than 200 and mitochondrial percentage exceeding 25. Raw RNA UMI counts of the aggregated data were normalized, followed by principal component analysis (PCA). The dataset was visualized and explored using the uniform manifold approximation and projection (UMAP) method, a nonlinear dimensional reduction technique.

### 4.5. Spatial Transcriptomics Data Analysis of Breast Tumor Tissue

Spatial transcriptomics dataset generated in our previous study [55] and available via GEO Database under the accession number GSE242311 was used to investigate gene expression of *EPCAM*-negative and *EPCAM*-positive tumor cells in primary tumors of five BC patients (invasive carcinoma of nonspecific type, luminal A and B, stage I–IIA, grade 2–3). Samples were filtered, excluding genes with nonzero expression in fewer than 10 tissue spots and tissue spots with fewer than 200 filtered genes. The raw counts were normalized using the SCTransform [56] function with default parameters. The uniform manifold approximation and projection (UMAP) technique was then applied to the SCTransform-normalized counts, utilizing the first 30 principal components determined through principal component analysis (PCA). The results were visualized using the Seurat package.

### 4.6. Statistical Analysis

The data were analyzed using GraphPad Prism 10 (GraphPad Software, San Diego, CA, USA). The two-way ANOVA was used for multiple comparisons between dependent groups; the sample was also checked for outliers. *p* < 0.05 was considered statistically significant.

## 5. Conclusions

The failure to detect CTCs in nearly half of BCs is likely since EpCAM-negative CTCs remain outside the scope of the assay. This is due to the predominant use of technologies based on the isolation of EpCAM-positive CTCs. The obtained results showed that when EpCAM-negative CTCs are taken into account, they are present in almost all BC cases. Moreover, EpCAM^high^, EpCAM^low^, and EpCAM-negative CTCs were present in different ratios in each case. The main result of the study is the finding of significant protein and gene expression alterations associated with the absence or intensity of EpCAM expression. Despite some inconsistency in the frequencies of detection of individual stemness and EMT features based on gene and protein expression in tumor cells, it can be assumed that EpCAM^high^ CTCs are more likely to possess diverse stemness features and epithelial EMT phenotype than EpCAM-negative CTCs. The expression of CD133 is dominant in EpCAM^high^ CTCs. EpCAM-negative CTCs and tumor cells, unlike EpCAM^high^ CTCs, are also characterized by the presence of stemness features and a more frequent hybrid EMT phenotype, necessitating a closer study of their clinical significance.

The overexpression in EpCAM^high^ CTCs of genes responsible for synthetic function indicates high functional activity. However, in our opinion, we should not underestimate the pathogenetic significance of the largest proportion of EpCAM-negative CTCs. Firstly, there may be small subpopulations of cells with aggressive properties among them, and secondly, the expression of genes related to immune-inflammatory reactions, which we have detected, makes them candidates for participation in the formation of premetastatic niches. Therefore, a more thorough investigation is imperative to reveal the prognostic significance of EpCAM-negative and EpCAM^low^ CTCs.

## Figures and Tables

**Table 1:** Clinicopathological characteristics of BC patients depending on the proportions of CTCs with different EpCAM expression variants.

Parameter	Cluster 1	Cluster 2	Cluster 3
T1	21.4% (3/14)	0% (0/6)	21.4% (3/14)
T2	57.1% (8/14)	100% (6/6)	64.3% (9/14)
T3	7.1% (1/14)	0% (0/6)	0% (0/14)
T4	7.1% (1/14)	0% (0/6)	7.1% (1/14)
No data	7.1% (1/14)	0% (0/6)	7.1% (1/14)
N0	71.4% (10/14)	50.0% (3/6)	64.3% (9/14)
N1	14.3% (2/14)	50.0% (3/6)	21.4% (3/14)
N2	7.1% (1/14)	0% (0/6)	0% (0/14)
N3	0% (0/14)	0% (0/6)	7.1% (1/14)
No data	7.1% (1/14)	0% (0/6)	7.1% (1/14)
Luminal A	7.1% (1/14)	0% (0/6)	0% (0/14)
Luminal B (Her2−)	35.7% (5/14)	33.3% (2/6)	42.9% (6/14)
Luminal B (Her2+)	21.4% (3/14)	0% (0/6)	7.1% (1/14)
TN	28.6% (4/14)	33.3% (2/6)	42.9% (6/14)
Her2+	7.1% (1/14)	33.3% (2/6)	7.1% (1/14)
Grade 1 Grade 2 Grade 3	0% (0/14) 78.6% (11/14) 7.1% (1/14)	0% (0/6) 83.3% (5/6) 16.7% (1/6)	14.3% (2/14) 57.1% (8/14) 28.6% (4/14)

**Table 2:** Stem features in EpCAM-negative, EpCAM^low^, and EpCAM^high^ CTCs, %.

CTC Phenotype	EpCAM-Neg, % (1)	Frequency of Occurrence, % (a)	EpCAM^low^, % (2)	Frequency of Occurrence, % (b)	EpCAM^high^, % (3)	Frequency of Occurrence, % (c)	*p* Value
CD44+CD24−/CD133+	0.00 (0.00–4.81)	26.5 (9/34)	0.00 (0.00–0.00)	0 (0/34)	0.00 (0.00–10.63)	26.5 (9/34)	*p*_a-b_ = 0.0021 *p*_b-c_ = 0.0021
CD44+CD24−/ CD133−	0.00 (0.00–20.00)	38.2 (13/34)	0.00 (0.00–0.00)	0 (0/34)	0.00 (0.00–0.00)	5.9 (2/34)	*p*_2-1_ = 0.0541 *p*_a-c_ = 0.0026 *p*_a-b_ < 0.0001
NonCD44+CD24−/CD133+	18.33 (0.00–62.23)	64.7 (22/34)	0.00 (0.00–51.79)	26.5 (9/34)	88.75 (59.06–100.00)	91.2 (31/34)	*p*_3-1_ = 0.0032 *p*_3-2_ = 0.0001 *p*_a-b_ = 0.0032 *p*_a-c_ = 0.0174 *p*_b-c_ < 0.0001
NonCD44+CD24−/CD133−	30.30 (0.00–61.51)	64.7 (22/34)	0.00 (0.00–44.64)	26.5 (9/34)	0.00 (0.00–17.50)	29.4 (10/34)	*p*_3-1_ = 0.0159 *p*_a-b_ = 0.0032 *p*_a-c_ = 0.0071

Footnote: “NonCD44 +CD24−” includes CD44−CD24−, CD44+CD24+, and CD44−CD24+ CTCs.

**Table 3:** Expression of EMT markers in EpCAM-negative, EpCAM^low^, and EpCAM^high^ CTCs.

CTC Phenotype	EpCAM-Neg (1)	Frequency, % (a)	EpCAM^low^ (2)	Frequency, % (b)	EpCAM^high^ (3)	Frequency, % (c)	*p* Value
CK7/8 + E-cadh−PanCK+N-cadherin−	0.00 (0.00–0.00)	2.9 (1/34)	0.00 (0.00–0.00)	0 (0/34)	0.00 (0.00–0.00)	0 (0/34)	
CK7/8 + E-cadh−PanCK+N-cadherin+	0.00 (0.00–0.00)	0 (0/34)	0.00 (0.00–0.00)	0 (0/34)	0.00 (0.00–0.00)	0 (0/34)	
CK7/8 + E-cadh + PanCK +N-cadherin−	0.00 (0.00–0.00)	0 (0/34)	0.00 (0.00–0.00)	0 (0/34)	0.00 (0.00–0.00)	0 (0/34)	
CK7/8 + E-cadh+ PanCK +N-cadherin+	0.00 (0.00–0.00)	0 (0/34)	0.00 (0.00–0.00)	0 (0/34)	0.00 (0.00–0.00)	0 (0/34)	
CK7/8 + E-cadh−PanCK-N-cadherin−	0.00 (0.00–4.75)	26.5 (9/34)	0.00 (0.00–0.00)	0 (0/34)	0.00 (0.00–0.00)	0 (0/34)	*p*_a-b_ = 0.0021 *p*_a-c_ = 0.0021
CK7/8 + E-cadh−PanCK− N-cadherin+	0.00 (0.00–0.00)	20.6 (7/34)	0.00 (0.00–0.00)	5.9 (2/34)	0.00 (0.00–0.00)	5.9 (2/34)	
CK7/8 + E-cadh+ PanCK− N-cadherin−	0.00 (0.00–0.00)	20.6 (7/34)	0.00 (0.00–0.00)	17.6 (6/34)	0.00 (0.00–0.00)	8.8 (3/34)	
CK7/8 + E-cadh+ PanCK− N-cadherin+	0.00 (0.00–0.00)	5.9 (2/34)	0.00 (0.00–0.00)	5.9 (2/34)	0.00 (0.00–0.00)	5.9 (2/34)	
CK7/8− E-cadh−PanCK+N-cadherin−	54.78 (0.00–85.71)	70.6 (24/34)	0.00 (0.00–4.16)	23.5 (8/34)	0.00 (0.00–25.89)	44.1 (15/34)	*p*_a-b_ = 0.0002 *p*_a-c_ = 0.0490 *p*_2-1_ = 0.0159
CK7/8−E-cadh−PanCK+N-cadherin+	0.00 (0.00–6.48)	35.3 (12/34)	0.00 (0.00–0.00)	2.9 (1/34)	0.00 (0.00–0.00)	0 (0/34)	*p*_a-b_ = 0.0012 *p*_a-c_ = 0.0002
CK7/8−E-cadh+ PanCK+N-cadherin−	0.00 (0.00–7.98)	26.5 (9/34)	0.00 (0.00–0.00)	8.8 (3/34)	0.00 (0.00–0.00)	0 (0/34)	*p*_a-c_ = 0.0021
CK7/8−E-cadh+ PanCK+N-cadherin+	0.00 (0.00–0.18)	23.5 (8/34)	0.00 (0.00–0.00)	2.9 (1/34)	0.00 (0.00–0.00)	0 (0/34)	*p*_a-b_ = 0.0272 *p*_a-c_ = 0.0049
CK7/8−E-cadh−PanCK− N-cadherin−	-	-	-		0.00 (0.00–25.89)	47.1 (16/34)	
CK7/8−E-cadh−PanCK− N-cadherin+	-	-	-		0.00 (0.00–10.63)	29.4 (10/34)	
CK7/8−E-cadh+ PanCK− N-cadherin−	-	-	-		8.33 (0.00–57.92)	52.9 (18/34)	
CK7/8−E-cadh+ PanCK− N-cadherin+	-	-	-		0.00 (0.00–0.00)	11.8 (4/34)	

**Table 4:** Frequency of detection of CTCs with E-cadherin or N-cadherin expression variants.

CTCs Phenotype	EpCAM-Negative (1)	EpCAM^low^ (2)	EpCAM^high^ (3)	*p* Value
E-cadherin+	47.1 (16/34)	26.5 (9/34)	8.8 (3/34)	*p*_3-1_ = 0.0009
N-cadherin+	55.9 (19/34)	8.8 (3/34)	5.9 (2/34)	*p*_2-1_ = 0.0001 *p*_3-1_ = 0.0000
E-cadherin+ N-cadherin+	29.4 (10/34)	8.8 (3/34)	5.9 (2/34)	*p*_3-1_ = 0.0230

**Table 5:** Comparison of the proportion and frequencies of tumor cells with different EpCAM expression among CTCs and in the primary tumor.

	EpCAM-Negative		EpCAM^low^		EpCAM^high^	
	Frequency, %	Proportion, %	Frequency, %	Proportion, %	Frequency, %	Proportion, %
CTCs	100 (11/11)	75.68 (38.46–85.71)	45.5 (5/11)	0.00 (0.00–10.00)	90.9 (10/11)	20.00 (7.69–61.54)
Primary tumor	90.9 (10/11)	15.33 (10.67–89.33)	90.9 (10/11)	38.00 (1.33–58.00) *p* = 0.0371	72.7 (8/10)	10.00 (0.00–33.33)

**Table 6:** Expression of stemness and EMT-related genes in *EPCAM*-negative and *EPCAM*-positive CTCs of BC patients.

Gene	EPCAM−CTCs	EPCAM+CTCs	*p* Value
KRT7	1.3% (3/228)	8.3% (1/11)	
KRT8	70.2% (160/228)	25.0% (3/11)	*p* = 0.02
KRT18	41.7% (95/228)	25.0% (3/11)	
Stemness-related genes			
CD44/CD24	5.3% (12/228)	8.3% (1/11)	
ALDH1A1	0.4% (1/228)	33.3% (4/11)	*p* < 0.0001
PROM1 (CD133)	0.4% (1/228)	25.0% (3/11)	*p* = 0.0004
POU5F1 (OCT4)	0.4% (1/228)	16.7% (2/11)	*p* = 0.0067
KLF4	1.8% (4/228)	16.7% (2/11)	*p* = 0.0100
SOX2	0.0% (0/228)	0.0% (0/11)	
MYC	0.4% (1/228)	16.7% (2/11)	*p* = 0.0067
Epithelial–mesenchymal transition genes			
CDH1	0.0% (0/228)	16.7% (2/11)	*p* = 0.0023
CDH2	0.4% (1/228)	0.0% (0/11)	
VIM	18.0% (41/228)	66.7% (8/11)	*p* = 0.0005
ZEB1	0.4% (1/228)	25.0% (3/11)	*p* = 0.0004
ZEB2	19.7% (45/228)	50.0% (6/11)	*p* = 0.005
SNAI1	0.0% (0/228)	8.3% (1/11)	
SNAI2	0.0% (0/228)	8.3% (1/11)	
TWIST	0.0% (0/228)	8.3% (1/11)	
FN1	0.4% (1/228)	8.3% (1/11)	
SCGB2A (mammoglobin A)	1.8% (4/228)	8.3% (1/11)	

**Table 7:** Expression of stemness and EMT-related genes in *EPCAM*-negative, *EPCAM* ^low^, and *EPCAM* ^high^ tumor spots in primary tumor of BC patients.

Gene	EPCAM-Negative (1)	EPCAM^low^ (2)	EPCAM^high^ (3)	*p* Value
KRT7	69.1% (1037/1500)	66.0% (1037/1571)	76.7% (1420/1851)	*p*_3-1_ < 0.0001 *p*_2-3_ < 0.0001
KRT8	95.9% (1438/1500)	97.9% (1538/1571)	99.9% (1849/1851)	*p*_2-1_ = 0.0012 *p*_3-1_ < 0.0001 *p*_2-3_ < 0.0001
KRT18	93.2% (1398/1500)	95.9% (1507/1571)	98.5% (1823/1851)	*p*_2-1_ = 0.0010 *p*_3-1_ < 0.0001 *p*_2-3_ < 0.0001
Stemness-related genes				
CD44/CD24	6.7% (100/1500)	1.2% (18/1571)	0.3% (5/1851)	*p*_2-1_ < 0.0001 *p*_3-1_ < 0.0001 *p*_2-3_ = 0.0025
ALDH1A1	9.4% (141/1500)	6.3% (99/1571)	3.6% (67/1851)	*p*_2-1_ = 0.0015 *p*_3-1_ < 0.0001 *p*_2-3_ = 0.0003
PROM1 (CD133)	7.7% (115/1500)	16.6% (261/1571)	52.4% (970/1851)	*p*_2-1_ < 0.0001 *p*_3-1_ < 0.0001 *p*_2-3_ < 0.0001
POU5F1 (OCT4)	1.1% (17/1500)	1.6% (25/1571)	2.5% (46/1851)	*p*_3-1_ = 0.0046
KLF4	25.5% (383/1500)	20.3% (319/1571)	27.6% (511/1851)	*p*_2-1_ = 0.0006 *p*_2-3_ < 0.0001
SOX2	0.3% (4/1500)	0.2% (3/1571)	0.0% (0/1851)	*p*_3-1_ = 0.0401
MYC	65.0% (975/1500)	80.8% (1269/1571)	95.6% (1770/1851)	*p*_2-1_ < 0.0001 *p*_3-1_ < 0.0001 *p*_2-3_ < 0.0001
Epithelial–mesenchymal transition genes				
CDH1	81.4% (1221/1500)	93.0% (1461/1571)	98.0% (1814/1851)	*p*_2-1_ < 0.0001 *p*_3-1_ < 0.0001 *p*_2-3_ < 0.0001
CDH2	4.5% (67/1500)	4.9% (77/1571)	3.6% (66/1851)	
VIM	95.7% (1435/1500)	96.2% (1511/1571)	96.3% (1783/1851)	
SNAI1	2.4% (41/1500)	4.1% (65/1571)	5.9% (109/1851)	*p*_2-1_ = 0.0376 *p*_3-1_ < 0.0001 *p*_2-3_ = 0.0233
SNAI2	51.2% (768/1500)	52.1% (819/1571)	58.1% (1075/1851)	*p*_3-1_ < 0.0001 *p*_2-3_ = 0.0006
ZEB1	33.6% (505/1500)	29.2% (458/1571)	26.1% (484/1851)	*p*_2-1_ = 0.0073 *p*_3-1_ < 0.0001
ZEB2	27.3% (409/1500)	25.1% (394/1571)	34.1% (631/1851)	*p*_3-1_ < 0.0001 *p*_2-3_ < 0.0001
TWIST	7.1% (106/1500)	5.8% (92/1571)	5.2% (97/1851)	*p*_3-1_ = 0.0290
FN1	99.1% (1486/1500)	100.0% (1571/1571)	100.0% (1851/1851)	*p*_2-1_ < 0.0001 *p*_3-1_ < 0.0001
SCGB2A2 (mammoglobin A)	29.1% (437/1500)	13.5% (212/1571)	2.32% (43/1851)	*p*_2-1_ < 0.0001 *p*_3-1_ < 0.0001 *p*_2-3_ < 0.0001

**Table 8:** Clinicopathological characteristics of BC patients.

Parameter		
Age	<35	8.82% (3/34)
	35–50	58.82% (20/34)
	>50	32.36% (11/34)
Tumor size (T)	1	20.59% (7/34)
	2	70.59% (24/34)
	3	2.94% (1/34)
	4	5.88% (2/34)
Stage	I	20.59% (7/34)
	IIA	50.00% (17/34)
	IIB	20.59% (7/34)
	IIIA	2.94% (1/34)
	IIIB	2.94% (1/34)
Molecular subtype	Luminal A	2.94% (1/34)
	Luminal B (HER2−)	38.25% (13/34)
	Luminal B (HER2+)	11.76% (4/34)
	Triple negative	35.29% (12/34)
	HER2-enriched	11.76% (4/34)
Lymph node metastasis	Yes	32.35% (11/34)
	No	67.65% (23/34)

## Data Availability

Dataset available on request from the authors.

## Supplementary Materials

The following supporting information can be downloaded at: https://www.mdpi.com/article/10.3390/ijms252011109/s1.
